# An aggregate analysis of many predicted structures to reduce errors in protein structure comparison caused by conformational flexibility

**DOI:** 10.1186/1472-6807-13-S1-S10

**Published:** 2013-11-08

**Authors:** Brian G Godshall, Yisheng Tang, Wenjie Yang, Brian Y Chen

**Affiliations:** 1 Department of Computer Science and Engineering, Lehigh University, Bethlehem, PA, 18015, USA

## Abstract

**Background:**

Conformational flexibility creates errors in the comparison of protein structures. Even small changes in backbone or sidechain conformation can radically alter the shape of ligand binding cavities. These changes can cause structure comparison programs to overlook functionally related proteins with remote evolutionary similarities, and cause others to incorrectly conclude that closely related proteins have different binding preferences, when their specificities are actually similar. Towards the latter effort, this paper applies protein structure prediction algorithms to enhance the classification of homologous proteins according to their binding preferences, despite radical conformational differences.

**Methods:**

Specifically, structure prediction algorithms can be used to "remodel" existing structures against the same template. This process can return proteins in very different conformations to similar, objectively comparable states. Operating on close homologs exploits the accuracy of structure predictions on closely related proteins, but structure prediction is often a nondeterministic process. Identical inputs can generate subtly different models with very different binding cavities that make structure comparison difficult. We present a first method to mitigate such errors, called "medial remodeling", that examines a large number of predicted structures to eliminate extreme models of the same binding cavity.

**Results:**

Our results, on the enolase and tyrosine kinase superfamilies, demonstrate that remodeling can enable proteins in very different conformations to be returned to states that can be objectively compared. Structures that would have been erroneously classified as having different binding preferences were often correctly classified after remodeling, while structures that would have been correctly classified as having different binding preferences almost always remained distinct. The enolase superfamily, which exhibited less sequential diversity than the tyrosine kinase superfamily, was classified more accurately after remodeling than the tyrosine kinases. Medial remodeling reduced errors from models with unusual perturbations that distort the shape of the binding site, enhancing classification accuracy.

**Conclusions:**

This paper demonstrates that protein structure prediction can compensate for conformational variety in the comparison of protein-ligand binding sites. While protein structure prediction introduces new uncertainties into the structure comparison problem, our results indicate that unusual models can be ignored through an analysis of many models, using techniques like medial remodeling. These results point to applications of protein structure comparison that extend beyond existing crystal structures.

## Introduction

Algorithms that compare protein structures generally represent proteins as rigid objects. This simplifying assumption can overlook related proteins in different conformations, but it enables the geometric similarity between two atomic structures to be rapidly measured [1,2]. Efficiency is crucial for most tools, which search large databases of protein structures for proteins with remote evolutionary relationships [3-8] or similar functional sites [1,9-12]. In both cases, conformational changes can disrupt the significant structural similarity that is required to distinguish similar proteins from those that are similar by random chance [13-15].

Conformational flexibility also affects algorithms that detect structural influences on binding specificity [16]. Beginning with a family of proteins with aligned binding cavities, these algorithms find cavity subregions that are conserved, potentially to accommodate the same molecular fragment. They also identify varying subregions, which might encourage differing ligands to bind. Finding regions like these can point to steric influences on specificity [17,18]. But conformational changes, from sweeping backbone movements to subtle rotamer tweaks, can introduce variations that do not relate to binding preferences. For example, the kinking of an alpha helix can cause the lipid binding cavity of yellow lupine PR-10 to appear radically different from other PR-10 proteins, despite similar binding preferences [16,19]. Without compensating for the effects of flexibility, algorithms for detecting influences on specificity are exposed to a considerable source of potential error.

Fortunately, these errors can be diminished, as we observed earlier [20], by using structure prediction algorithms to *remodel *proteins into conformations that are more comparable. Remodeling designates one structure as a template against which to model the structures of other proteins, thereby reducing differences in backbone and sidechain conformation. This process can enable binding sites in closed or inactive conformations, which were previously not comparable, to be more accurately compared against other sites. When the proteins to be compared are closely related, as they frequently are when searching for influences on binding specificity, remodeling exploits the superior accuracy of structure prediction algorithms on close homologs [21,22].

But predicted structures are not generated deterministically. Variations in backbone and sidechain structure occur frequently between models generated from the same inputs. These variations are their own source of classification errors, and they limit the potential applicability of remodeling. Extending our earlier work, this paper examines the impact of variations from structure prediction on the comparison of protein binding cavities. We then evaluate how they affect the accurate detection of conserved and varying regions that influence binding specificity. Despite tremendous individual variations, an aggregate analysis of many predicted structures enabled us to make accurate comparisons that would have been less accurate.

## Related work

Conformational rigidity is an fundamental assumption in the design of almost every protein structure comparison algorithm. This assumption originates in the digital representations that algorithms use to represent whole protein structures for comparison. Geometric invariants (e.g. [3]), matrices of inter-point distances [5], and points in space [3,4,6,7,23], the most common representation, can faithfully represent atomic positions and types, but they do not describe atomic motion.

To permit larger differences in protein structure, a second category of point-based representations limit the comparison of protein structures to binding sites alone, enabling the rest of the structure to change. These binding site "motifs" represent catalytic sites [1,9,10,24], evolutionarily significant amino acids [2], "pseudo-centers" of protein-ligand interactions [25], and "pseudoatoms" on amino acid sidechains [26]. These representations tolerate infinite variation outside the binding site, in order to rapidly scan databases of protein structure (e.g. the PDB [27]) and identify proteins with very different evolutionary origins but similar functional sites.

All comparison algorithms tolerate a limited degree of structural variation. This tolerance is typically achieved by requiring corresponding atoms in aligned structures to fall within a maximum distance. Distance criteria can tolerate small conformational changes, but larger variations, especially bond rotations that can lever one part of the protein away from another, can cause similar proteins to seem dissimilar.

Further relaxation of rigidity assumptions requires algorithms that have specialized representations of protein structures that can detect related proteins in very different conformations. Flexprot [28] and FlexSnap [29], for example, use virtual hinges connecting rigid components to represent protein structures, permitting similar proteins in altered conformations to still be aligned. Another approach, Posa [30], detects similar molecular components in different conformations using partial order graphs. These kinds of representations, which have inbuilt accommodations for structural variation, can enable the detection of proteins with very remote evolutionary similarities.

But many phenomena depend on the ability of closely related proteins to selectively bind different ligands. This paper examines protein binding sites with Boolean set operations [16] to find similarities [17] and variations [18] that cause differences in binding preferences. Comparisons with Boolean set operations are just as sensitive to conformational change as existing methods, as we observed on the START domains [16], but the analysis of similar proteins has specific advantages: It may be possible to predict the structures of closely related proteins in order to return them to a comparable conformation, as we observed earlier [20], because homology modeling algorithms are extremely accurate on similar proteins [21,22]. Also, remodeling does not require representations of protein structures that have specific features that compensate for for conformational change. This paper extends our earlier work by examining the novel challenges and potentials intrinsic to the remodeling strategy.

## Methods

In earlier work [20], we described a hybrid method for remodeling protein structures that enables binding sites to be compared even when major conformational changes obscure the binding site. We paraphrase this work, an integration of several existing methods, below. We then extend our remodeling approach by describing how multiple predicted structures can be used to compensate for variations in structure predictions. We refer to this new process as *Medial Remodeling*, to contrast from our earlier approach, which we will call *Simple Remodeling*.

### Simple remodeling

Remodeling is a preprocessing step for the comparison of two closely related protein structures *A *and *B*. These structures have the same fold and biological function, but exhibit different binding preferences. Both structures are assumed to exhibit a range of backbone or side chain conformations. While some conformations of *B *may be inactive or otherwise unusual, the purpose of remodeling is to return the backbone and sidechain conformation of *B *to a state that is as similar to that of *A *as possible, so that their binding sites can be objectively compared.

When we remodel *B *onto *A*, we designate *A *as a structural template and the amino acid sequence of *B *as a query sequence, to build a new model of *B*. Since *A *is the template, the amino acids of *B *will be positioned to resemble the backbone topology and sidechain orientations of *A*. With the understanding that this application is focused on closely related proteins, we presume that the resulting model of *B *will be in a conformation that is more similar to that of *A*, and that their binding sites will be comparable, while they may not have been before.

**Model building**. We use NEST for model building [31]. Input for NEST is the template structure *A *and an alignment of the amino acid sequences of *B *and *A*, generated with Clustalw [32]. With these inputs, and otherwise default parameters, NEST builds models via "artificial evolution": NEST iteratively modifies the template structure with insertions, deletions, and mutations from the sequence alignment. After every modification, relaxation steps minimize van der Waals, hydrophobic, electrostatic, torsional, and hydrogen bonding potentials, and only the most energetically favorable modification is accepted in each iteration. Once the template has been completely changed into the query protein, the model is returned as output. This process is nondeterministic, creating variations in predicted structures.

**Structural alignment**. Once the model structure is generated, we superpose the model onto the template using Ska [23], an algorithm for whole-structure superposition. The superposed model of *B *is now said to be remodeled onto *A*.

### Representation and comparison

**Figure 1:**
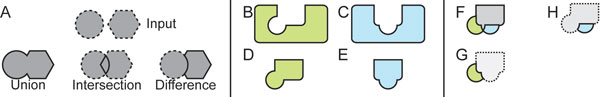
**Boolean Set Operations**. Boolean Set Operations (a) on solid representations of protein structures (b,c) or aligned binding sites (d,e,f) can be used to detect similarities in binding sites (g) that accommodate the same molecular fragment or variations (h,i) that cause differences in specificity [16-18].

**Binding cavity representation**. We use VASP [16] to generate and compare solid representations of binding cavities, using Boolean set operations (Figure 1a). As input, we begin with the structure of a protein *C *and the putative position of a bound ligand. Typically this position is determined by superposing *C *onto the structure of a similar protein in complex with a ligand. In our data set, we used the atrolactic acid bound to pseudomonas putida mandelate racemase in pdb structure 1mdr to generate cavities in the enolase structures. Among tyrosine kinase structures, we used the staurosporine bound to human Abelson kinase (pdb structure 2hz4).

After superposition, the ligand in the complex overlaps the approximate location of the binding site in the aligned structure. We use it's position and shape to define a solid representation of the binding cavity in the aligned structure: First, we create a series of spheres with a 5 Å radius, centered at each ligand atom. We then calculate the Boolean union of the spheres. Second, using the Trollbase library from GRASP2 [7], we calculate a molecular surface of the model. Trollbase generates a closed surface using the classic rolling-probe technique [33,34] with a water-sized probe of radius 1.4 Å, which we interpret as a volumetric solid. Using VASP, we compute the Boolean difference between the molecular surface from the union of spheres. Third, using a probe of radius 5.0 Å, we again use the Trollbase library to create an "envelope surface", based on external cavity boundaries used in SCREEN [35]. Finally, with VASP, we calculate the Boolean intersection between what remains of the ligand spheres and the envelope surface. The resulting region is a solid representation of the binding cavity on the model structure. This technique was described earlier, in [16]. After this procedure is complete, a solid representation of the binding cavity is ready for comparison.

**Binding site comparison**. Two binding cavities are structurally different if there exists a region within one binding cavity that is not within the other. Regions like these could potentially accommodate molecular fragments in one cavity that would not fit in the other cavity. We can identify and measure differences like these between two cavities *A *and *B *by computing the volume of the largest contiguous region where the two cavities do not overlap (e.g. Figure 1h,i). We call this region the largest *fragment *between *A *and *B*. Similar cavities tend to exhibit fragments with very small volumes, while cavities with different binding preferences exhibit larger fragments [18].

Between *A *and *B*, we find the largest fragment by first generating the symmetric Boolean difference (*A *- *B*) ∪ (*B *- *A*). This process creates several fragments, because *A *and *B *are never identical. We then isolate every fragment by using a graph-based technique that we developed earlier [18,36]. Next, we compute the volume of every fragment using the Surveyor's Formula [16,37]. Finally, we return the fragment with the largest volume (in Å^3^). We use its volume as a proxy for the degree of similarity between *A *and *B*: Large fragment volumes indicate substantial differences in cavity shape, while small fragment volumes indicate similarities in cavity shape.

**Figure 2:**
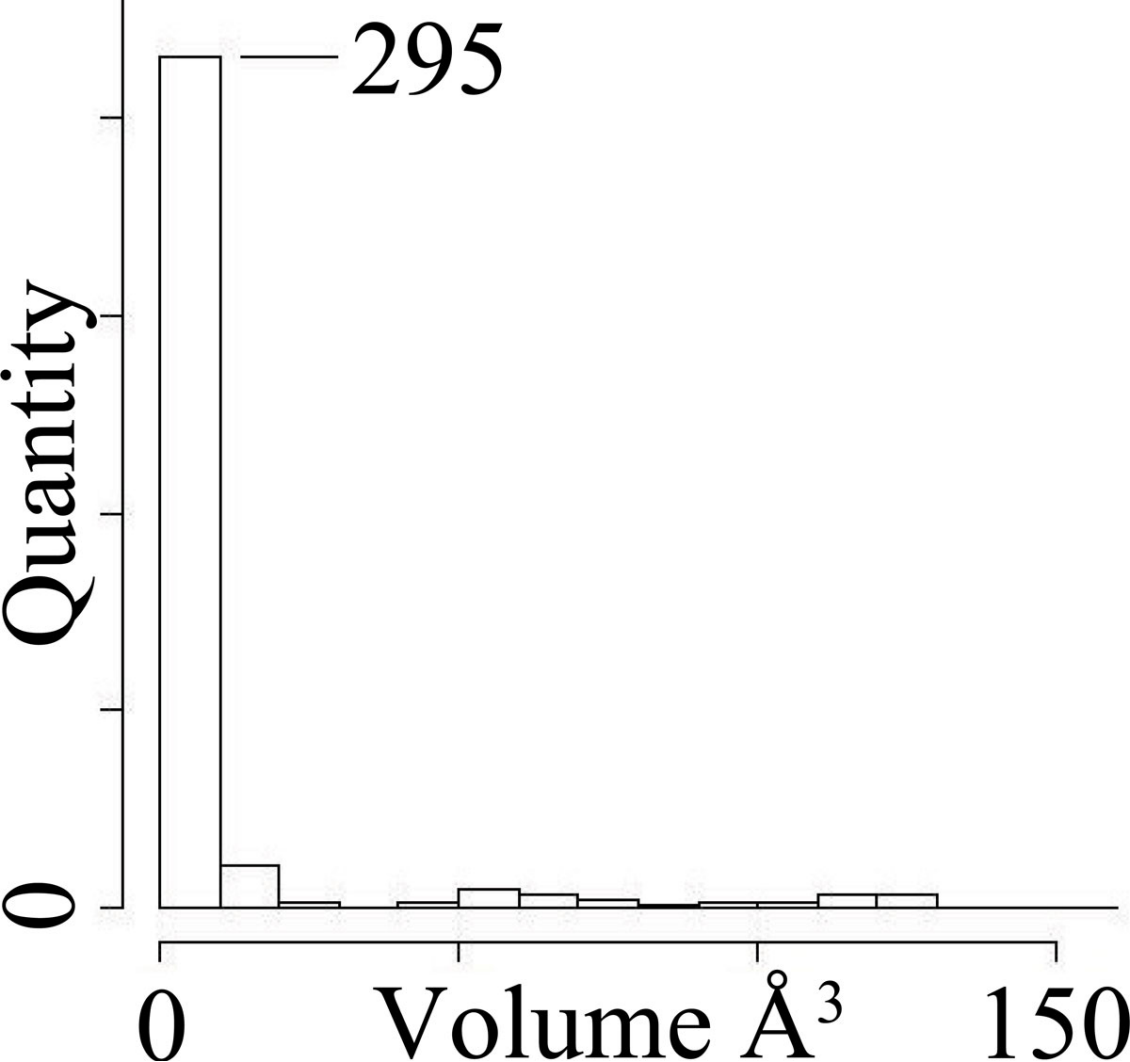
**Histogram of fragment volumes between enolases with identical binding preferences**. 295 out of the 340 plotted here occupied less than 10 Å^3 ^in spatial volume.

**Statistical modeling**. Fragments observed between cavities with identical binding specificity are generally very small, because the binding cavities are very similar. For example, among enolases that have similar binding preferences (Table 1), 248 out of 340 fragments occupied less than one cubic angstrom. 295 fragments occupied less than 10 Å^3 ^(Figure 2). Tyrosine kinases exhibited similar distributions.

**Table 1:** PDB codes of structures used.

**Enolase Superfamily (homogeneous):** **Enolases: **1e9i, 1iyx, 1pdy, 2pa6, 2xsx, 2xsx **Enolase Superfamily (homogeneous, redundant):** **Enolases: **1ebh, 1els, 1nel, 2al2, 3enl, 7enl, 1te6, 1ebg, 1one **Tyrosine Kinases (homogeneous):** **Small Gatekeeper residue: **1qcf, 1fgi, 1fpu, 1fvr, 1gjo, 1irk, 1k2p, 1m14, 1m7n, 1qpc, 1r0p, 1t45, 1u4d, 1yvj, 1ywn, 2src **Tyrosine Kinases (homogeneous, redundant):** **Small Gatekeeper residue: **2hz4, 2e2b, 2hyy, 2hz0, 2hzn, 2hzi, 2xyn, 2hmi, 3kf4, 3kfa, 3ms9, 3mss **Enolase Superfamily (heterogeneous):** **Enolases: **1e9i, 1ebh, 1iyx, 1pdy, 1te6, 2pa6, 2xsx, 3otr, **Mandelate Racemase: **2ox4, **Muconate Lactonizing Enzyme: **2pgw, 2zad **Tyrosine Kinases (heterogeneous):** **Small Gatekeeper residue: **2hz4 **Large Gatekeeper residue: **1fvr, 1luf, 1rjb, 1sm2, 1snu, 1snx

Bolded structures were selected as templates. Italicized structures have closed or inactive conformations.

This pattern of fragment volumes, a characteristic abundance of small values, can be approximated closely by the log-normal distribution [18,36], which allows us to estimate the value of the distribution at any point on the positive × axis. In particular, we can estimate the probability *p *of observing a hypothetical fragment with volume equal to or larger than that of a given fragment. When *p*, often referred to as the *p*-value, is less than .05, it is called *statistically significant*.

Since we model distributions of fragment volumes from cavities with identical binding preferences, the *p*-value is an estimate of the probability of observing a fragment of a given size between cavities with identical binding preferences. If the *p*-value is too low, e.g. less than 0.05, then we reject our assumption as improbable, and predict the logical opposite: that the cavities must have different binding preferences. This prediction is based on our evaluation of the data, and not a statement of fact.

This work uses statistical modeling to evaluate the pattern of fragment volumes observed between unmodeled and modeled cavities. To determine the effect of remodeling on fragment volume, we use unmodeled cavities to train our statistical model, as we have in earlier work [17,18,36,38]. This approach enables use to measure the improvement in prediction accuracy that can be achieved in remodeled structures, in comparison unmodeled structures.

### Compensating for variations in predicted structures

Protein structures are predicted by generating a range of plausible models and selecting the highest scoring model. As a result, separate prediction efforts generate different models. In our experimentation, we have observed that variations in the models generated, using the same template and query sequence, can lead to differences in the shape of predicted binding cavities that are hundreds of cubic angstroms in volume, while other template-sequence pairs differed insubstantially. Rather than evaluating the accuracy of the model, a topic that is well studied in other fields, we seek to avoid extreme conformations through sampling.

To make our simple remodeling process of a protein *B *onto *A *more dependable, medial remodeling generates a model of *B *100 times. For each of the 100 models, we compute the largest fragment between each remodeled binding site and the binding site of *A*, and measure its volume. Finally, we use the median of these volumes to approximate the structural difference between the binding sites of *B *and *A*.

The median of fragment volumes eliminates the effect of extreme values that can occur from erroneous models. Which such models are generated rarely, their effect can create erroneously defined binding cavities that differ from the actual binding site by thousands of cubic angstroms. In our experimentation, we observed that template-query pairs that created model binding cavities with relatively small variations still exhibited extremal cases.

### Data set construction

**Protein families**. We used the enolase superfamily and the tyrosine kinases to test the effectiveness of our methods. We chose these superfamilies because both superfamilies are the subject of considerable study, which enables us to use established experimental evidence to evaluate the accuracy of our computational predictions. In addition, publicly available structures of both superfamilies demonstrate changes in binding site conformation that have well known functional impacts. Finally, uncontroversial classifications separate both superfamilies into subfamilies with different binding preferences based on well documented features in the binding site. These macroscopic criteria ensure that we can realistically test our methods and verify the accuracy of our results.

Conformational flexibility at the binding site is essential for function in both superfamilies. A flexible "capping domain" affects specificity in the enolase superfamily [39], enabling the active site to close. In the tyrosine kinases, multiple configurations of the phosphorylation loop (the "P-loop") [40] and the "DFG flip" on the activation loop [41] have direct effects on catalytic activity. These conformational changes interfere with the detection of specificity determinants in enolases and tyrosine kinases by radically altering the shape of the binding site.

The enolase superfamily catalyzes reactions that abstract a proton from a carbon adjacent to a carboxylic acid. These reactions occur near the C-terminal ends of beta sheets in a conserved TIM-barrel, where amino acids act as acid/base catalysts to facilitate several different reactions [42]. Our experimentation examines the differences in specificity between three enolase subfamilies. The first subfamily (EC 4.2.1.11), also known as enolases, catalyze the dehydration of 2-phospho-D-glycerate to phosphoenolpyruvate [43]. Mandelate racemases, the second subfamily (EC 5.1.2.2), convert (R)-mandelate to and from (S)-mandelate [44]. The third subfamily, muconate-lactonizing enzymes (EC 5.5.1.1), catalyze the reciprocal cycloisomerization of cis,cis-muconate and muconolactone in muconate-lactonizing enzyme [42].

Tyrosine kinases (EC classes 2.7.10.1 and 2.7.10.2) transfer phosphate groups from adenosine triphosphate to a tyrosine sidechain on an acceptor protein. This superfamily performs essential functions in cell signalling (e.g. [45]), and because of their central role in cell death and cell growth, tyrosine kinases are frequent targets in anticancer inhibitor design (e.g. [46,47]). Since tyrosine kinases are involved in a diverse spectrum of activities, their binding specificity can be examined in many contexts, with different acceptor proteins, with different drugs, and more. For the purposes of testing our method, our experimentation targets only the impact of the *gatekeeper *residue [48] on inhibitor specificity: Kinases with small gatekeeper residues can be targeted by many drugs, while those with large gatekeeper residues exhibit resistance to a broad spectrum of inhibitors [49]. Focusing on the ATP/inhibitor binding site creates larger categories that are more suitable for prediction testing and statistical modeling than finer classifications that consider both the inhibitor binding site and the acceptor binding interface.

**Selection**. From the enolase and tyrosine kinase superfamilies, we generated two datasets. The first dataset, called the *homogenous dataset*, is composed of an enolase subdivision and a tyrosine kinase subdivision where every member of the same subdivision has similar binding preferences. The second dataset, the *heterogeneous dataset*, contained different subdivisions with different binding preferences. Both datasets were initially created using sequentially nonredundant structures. Using Clustalw [32], we evaluated pairwise sequence identity and eliminated one member of any pair with greater than 90 percent identity. Afterwards, several conformationally different but sequentially redundant structures were added to the homogeneous dataset, to guarantee conformational diversity. These additional structures were deliberately selected to exhibit conformational differences described as "closed", "partially closed" or "inactive".

After structure selection, one or more members of each data set were chosen to serve as modeling templates. The sequences of the remaining structures were remodeled to the template with NEST [31], and then structurally aligned to the template using Ska [23]. The same structures, without remodeling, were aligned to the template but not modeled, for use as a control set. Binding cavities in all structures were generated using the method described above.

Our criteria for selecting modeling templates was based on the presence of a ligand in the template. This ligand was used to define a cavity in the template and all aligned models. The presence of a bound ligand further confirms the conformation of the binding site as being able to bind other molecules.

## Experimental results

In earlier work [20], we demonstrated that simple remodeling on protein structures that exhibit the same function and binding preferences but different conformations can enable them to be more accurately compared. We also showed that proteins with binding preferences that are different from the template do not become indistinguishable from proteins with binding preferences that are the same as the template after simple remodeling. Here, we reconfirm these earlier results using Clustalw to align the query sequence to the template, rather than structure alignments, used earlier. We then extend our earlier work by demonstrating the range of cavity variations that can be observed by medial remodeling, and finally illustrating how medial remodeling can isolate variations in cavity shape that relate to differences in specificity despite the nondeterministic nature of structure prediction.

### Simple remodeling on proteins with homogeneous binding preferences

**Figure 3:**
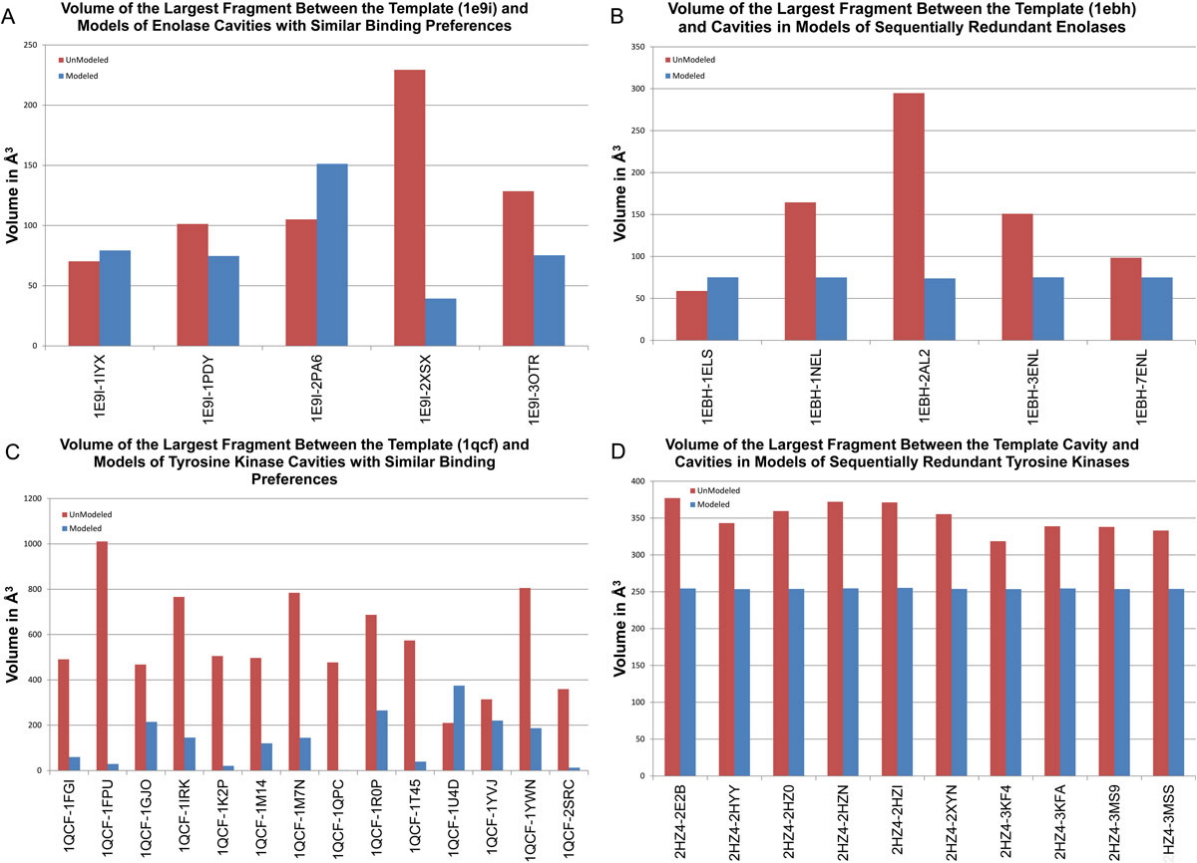
**Fragment volumes from cavities with similar binding preferences, before and after remodeling**. Red bars indicate the volume of the largest fragment between the template cavity and cavities with similar binding preferences, before remodeling. Blue bars indicate the volume of the largest fragment between the template cavity and cavities with similar binding preferences, after remodeling. Sequentially nonredundant (A) and redundant (B) enolases are shown on the top. Sequentially nonredundant (C) and redundant (D) tyrosine kinases are shown on the bottom.

We remodeled all sequentially nonredundant members of the homogeneous enolase dataset onto the structure of saccharomyces cerevisiae enolase (1ebh). The sequentially nonredundant members of the homogeneous tyrosine kinase dataset were remodeled onto the structure of homo sapiens haematopoetic cell kinase (1qcf). A comparison of the volumetric differences between modeled and unmodeled cavities (Figure 3) revealed distinct differences: 4 out of 5 enolase cavities and 13 out of 14 tyrosine kinase cavities were more similar after remodeling then before remodeling. Figure 3a and 3c illustrate the degree of increased similarity among enolases and kinases, respectively. In almost all cases, remodeling proteins with similar binding preferences in different conformations yielded binding cavities that were more similar than before.

To evaluate the impact of conformational change independent of sequence differences, we also remodeled structures of enolases and tyrosine kinases that had substantial conformational differences but greater than 90% sequence identity with their respective template (Figure 3b,d). Among redundant enolases, the largest fragment had reduced volume in 4 out of 5 cases. The largest fragment between redundant kinases had reduced volume in 10 out of 10 cases. Because the sequences modeled were very similar, the cavities modeled were also very similar and they exhibited fragments with the template cavity of a similar size. When sequence identity is very high, remodeling frequently enhanced cavity similarity.

To evaluate how remodeling can assist in the detection of cavities with similar binding preferences, we built statistical models of fragment volume between enolases and tyrosine kinases with similar binding preferences. Before remodeling, the volume of the largest fragment between enolase cavities and their template was statistically significant in 40% of the data set. These cavities would have been incorrectly classified as having different binding preferences. After remodeling, the volume of the largest fragment was statistically significant in 20% of the dataset. Among tyrosine kinases, the volume of the largest fragment, between the tyrosine kinase cavities and their template, was statistically significant in 86% of the dataset. After remodeling, the volume of the largest fragment was statistically significant in just 7$ of the dataset. These results demonstrate that remodeling can reduce geometric dissimilarities related to conformational change that can that can cause similar binding sites to appear different and be incorrectly classified.

### Simple remodeling on proteins with heterogeneous binding preferences

**Figure 4:**
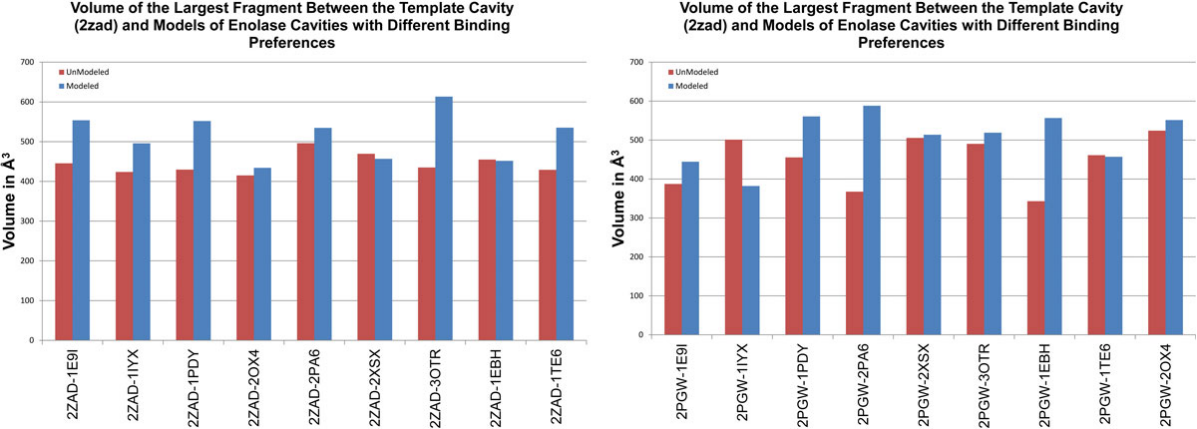
**Fragment volumes from enolase cavities with different binding preferences, before and after remodeling**. Red bars indicate the volume of the largest fragment between the template cavity and cavities with different binding preferences, before remodeling. Blue bars; after remodeling. The left graph plots results with enolase template 2pgw, the right with 2zad.

Simple remodeling of proteins with similar binding preferences may enhance the similarity of their binding sites and mitigate variations caused by conformational change, but in the context of comparing proteins with similar folds, simple remodeling could make proteins with different binding preferences appear too similar. To evaluate this possibility, we remodeled the members of the heterogeneous enolase superfamily against muconate cycloisomerase from Sinorhizobium meliloti (2pgw) and Thermotoga maritima (2zad). When modeling 2pgw as the template, the volume of largest fragment was greater after remodeling in 7 out of the 9 models and nearly identical in the remaining two. When using 2zad as a template, the volume of the largest fragment was greater in 7 out of the 9 models. These volumes are plotted in Figure 4. All of the largest fragments were statistically significant, and thus pointing to differences in binding preferences, relative to fragments between enolase proteins with similar binding preferences.

**Figure 5:**
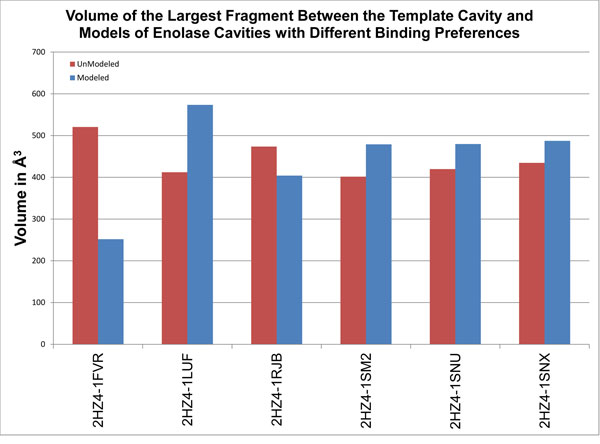
**Fragment volumes from tyrosine kinase cavities with different binding preferences, before and after remodeling**. Red bars indicate the volume of the largest fragment between the template cavity (2hz4) and cavities with different binding preferences, before remodeling. Blue bars indicate the volume of the largest fragment between the template cavity and cavities with different binding preferences after remodeling.

We also remodeled the tyrosine kinases with large gatekeeper residues using homo sapiens abelson kinase (2hz4) as a template. The largest fragment was larger after remodeling in 4 out of 6 models (Figure 5). All of the largest fragments were again statistically significant. Remodeling proteins onto templates with different binding preferences generally did not enhance similarity between the binding cavities of modeled proteins and the cavity in the template. In fact remodeling appears to frequently accentuate structural differences. These results suggest that, in the context of a general application, remodeling a set of proteins with both similar and different binding preferences may contribute to a more sensitive classification of proteins with different binding preferences.

### Binding site variations in structure predictions

**Figure 6:**
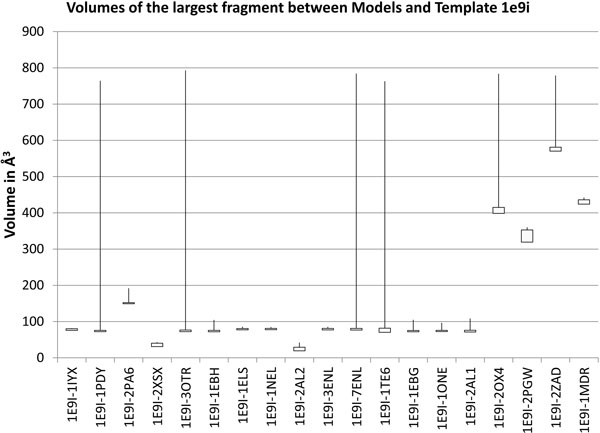
**Extreme values in modeled binding cavities**. Spectrum of the volumes of the largest fragment between the binding site of the template, E. coli enolase (pdb; 1e9i), and 100 models of proteins in the enolase dataset. The bottom of the rectangles in each column represent the volume of the fragment at the 25*^th ^*percentile of this spectrum, and the top of the rectangles represent the volume of the fragment at the 75*^th ^*percentile. Lines extending from the bottom of the box downwards and from the top of the box upwards end at the minimum and maximum volume observed for the largest fragment. While the weight of the distribution of volumes sometimes (though not always) fell within a narrow range, the maximum fragment volume was occasionally very high.

We performed medial remodeling on all dataset structures onto all template structures. For each of the 100 models generated between each template-sequence pair, we measured the volume of the largest fragment between the binding site of the model and that of the template. These volumes varied considerably between maximum and minimum, even when range between the 25*^th ^*percentile volume and the 75*^th ^*volume was very narrow. For example, among the enolases in our dataset, with the 1e9i template, the largest fragments were larger than 750 Å^3^, even though more than half of the largest fragments were approximately 75 Å^3 ^(Figure 6). With the 1ebh template, the largest fragments were larger than 850 Å^3^, though most of the largest fragments were approximately 75 Å^3^. Among the kinases, with the 1qcf template, the largest fragment was 1387 Å^3^, though most of the largest fragments were approximately 92 Å^3^. With the 2hz4 template, the largest fragment was 1438 Å^3^, and most of the largest fragments were approximately 342 Å^3^.

In isolation, the generation of a modeled structure with an unusual binding cavity is not very high. However, in the context of a structural classification effort, where many structures must be modeled, the probability of generating at least one unusual model increases with the size of the dataset, through multiple testing. By eliminating extrema, we hypothesize that medial remodeling can maintain accurate classification despite the nondeterministic nature of structure prediction.

### Evaluating medial remodeling

**Figure 7:**
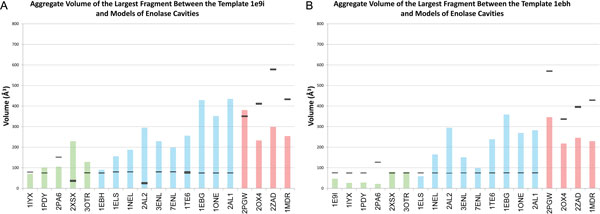
**Medial remodeling on enolase cavities**. Medial remodeling on a range of query sequences (horizontal axis) and the template. Black rectangles indicate volume of the largest fragment at the 25*^th ^*percentile of this spectrum, an the top of the rectangle represents that of the 75*^th ^*percentile. Colored bars indicate the volume of the largest fragment from the unmodeled query structure. Green bars indicate proteins that are sequentially nonredundant with the template, blue bars indicate proteins that are sequentially similar to the template, and red bars indicate proteins with different binding preferences.

We performed medial remodeling on all dataset structures onto all six template structures, computing the median of the volumes of the largest fragments in all 100 models. For dataset structures remodeled onto the enolase templates, 1e9i and 1ebh (Figure 7a,b) with similar binding preferences, the median volume of the largest fragment was never statistically significant, except in the case of 2pa6. The largest fragment computed with an unmodeled structure was larger, sometimes considerably larger, than the median volume, and larger still in cases of conformation change. In the case of 2XSX and most of the sequentially redundant enolase structures, the largest fragment from an unmodeled structure is statistically significant, and thus indistinguishable from proteins with different binding preferences. Among dataset structures with different binding preferences modeled onto 1e9i and 1ebh, the median of the largest fragment volume was always statistically significant and a conspicuous indicator of differing binding preferences. It is clearly possible that medial remodeling can permit enolases of different binding preferences to be distinguished, despite significant conformational differences, like a closed active site.

**Figure 8:**
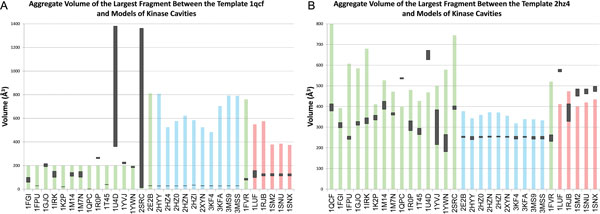
**Medial remodeling on tyrosine kinase cavities**. See legend from Figure 7, which presents the data in the same format.

For tyrosine kinase structures with small gatekeeper residues remodeled onto the templates 1qcf and 2hz4, the median volume of the largest fragment between the template and the model was frequently smaller than the fragment volume computed with queries from large gatekeeper amino acids (Figure 8a,b). The median volume of the largest fragments between the ATP binding cavity of 1qcf and all of the modeled ATP binding cavities of the large-gatekeeper tyrosine kinases were statistically significant, but the median volume of 11 of the modeled binding cavities from 26 tyrosine kinases were also statistically significant. Variations in tyrosine kinase binding cavities were much larger than among enolases, and the difficulty of this classification problem is apparent from the 11 incorrect predictions here. For example, the modeled kinase 2SRC exhibits cavities ranging from near zero to 1400 Å^3^, primarily because of the great diversity in models for 1T45 and 2SRC. Medial remodeling on tyrosine kinases based on the 2hz4 template produced similar results: Medial remodeling eliminated models where the binding cavity was extremely dissimilar, and, approximately half the time, models of cavities with similar binding preferences were more similar to the template than those with different binding preferences. Patterns of statistical significance revealed a similar trend.

These results, taken at a medium scale, suggest that medial remodeling can produce effective predictions, as in the enolase superfamily, but remodeling may not be as successful for very diverse superfamilies, like the tyrosine kinases. These results also demonstrate that median remodeling can eliminate structural outliers caused by the nondeterministic nature of structure prediction algorithms while reducing errors from conformational change.

## Conclusions

We have demonstrated simple and medial remodeling approaches for comparing binding sites in flexible proteins. While most algorithms for comparing protein structures are focused on the identification of remote homologs, we seek to predict structural determinants of specificity among closely related proteins. Our approach exploits the relatedness of our datasets by using structure prediction algorithms to compensate for conformational flexibility. Since homology modeling is most accurate when predicting structures that are similar, our approach strongly complements the intended application.

We demonstrated our results on sequentially nonredundant datasets representing the enolase and the tyrosine kinase superfamilies. Starting with similar structures in different conformations, we observed that simple remodeling onto templates with similar binding preferences could normalize differences in cavity shape. This approach enabled accurate comparisons even when the conformational differences were extreme, as in the case of inactive conformations.

In such cases, the improvement in prediction accuracy from remodeling can be significant. Remodeled enolases and tyrosine kinases that began with large conformational differences resulted in models that showed only statistically insignificant differences in shape. These differences were insufficient to cause proteins with similar binding preferences to be erroneously classified as having different binding preferences. In contrast, enolases and tyrosine kinases with different binding preferences exhibited binding cavities that remained dissimilar or became more dissimilar after remodeling. Remodeling enables structural similarities to be revealed among binding sites with similar binding preferences but does not make binding sites with different binding preferences indistinguishably similar.

Our results also demonstrate that simple remodeling is not uniformly successful in generating comparable models. When modeling structures from our diverse dataset of tyrosine kinases, some binding sites did not become as similar to the template as other sites, and in some cases they became very different. Due to the nondeterministic nature of structure prediction, unusual models are occasionally generated that exhibit very different cavities. We demonstrated that medial remodeling avoids the errors that might be generated by unusual models. Medial remodeling can be very successful, as it was on the enolase superfamily, in separating structures with very different binding preferences. In other applications, the differences identified through medial remodeling may not reach statistical significance.

These results illustrate a new approach to the comparison of protein structures that tolerates radical changes in molecular conformation. This approach highlights a new application for structure prediction, for the comparison of protein structures, that will support the detailed characterization of protein binding specificity: Protein structures in different conformations, due to differences in crystallographic method, different states of ligation or those in active or inactive conformations, can still be accurately compared. Protein sequences produced by high throughput sequencing technologies or gene resequencing efforts can be analyzed from a structural perspective. Together with other sources of biological data, volumetric analyses connected to homology modeling algorithms can offer important advancements to efforts in protein engineering, the study of drug resistance, and protein function annotation.

## Competing interests

The authors declare that they have no competing interests.

## Authors' contributions

B.G.G. and B.Y.C. developed the experiments and figures together. B.G.G. Ran the experiments. Y.T. and W.Y. ran the statistical analysis. B.G.G. and B.Y.C. wrote the paper.

## Declarations

The publication costs for this article were funded by the corresponding author.

This article has been published as part of *BMC Structural Biology *Volume 13 Supplement 1, 2013: Selected articles from the Computational Structural Bioinformatics Workshop 2012. The full contents of the supplement are available online at http://www.biomedcentral.com/bmcstructbiol/supplements/13/S1.

## References

[B1] Barker JA Thornton JM An algorithm for constraint-based structural template matching: application to 3D templates with statistical analysis Bioinformatics 2003 13 13 1644 1649 10.1093/bioinformatics/btg226 12967960

[B2] Chen BY Fofanov VY Kristensen DM Kimmel M Lichtarge O Kavraki LE Algorithms for structural comparison and statistical analysis of 3D protein motifs Pac Symp Biocomput 2005 13 334 45 15759639

[B3] Nussinov R Wolfson HJ Efficient detection of three-dimensional structural motifs in biological macromolecules by computer vision techniques Proc Natl Acad Sci USA 1991 13 23 10495 9 10.1073/pnas.88.23.10495 1961713 PMC52955

[B4] Orengo CA Taylor WR SSAP: sequential structure alignment program for protein structure comparison Methods Enzymol 1996 13 617 635 8743709 10.1016/s0076-6879(96)66038-8

[B5] Holm L Sander C Mapping the protein universe Science 1996 13 5275 595 603 10.1126/science.273.5275.595 8662544

[B6] Shindyalov IN Bourne PE Protein structure alignment by incremental combinatorial extension (CE) of the optimal path Protein Eng 1998 13 9 739 47 10.1093/protein/11.9.739 9796821

[B7] Petrey D Honig B GRASP2: visualization, surface properties, and electrostatics of macromolecular structures and sequences Methods Enzymol 2003 13 492 509 14696386 10.1016/S0076-6879(03)74021-X

[B8] Xie L Bourne PE Detecting evolutionary relationships across existing fold space, using sequence order-independent profile-profile alignments Proc Natl Acad Sci USA 2008 13 14 5441 6 10.1073/pnas.0704422105 18385384 PMC2291117

[B9] Russell RB Detection of protein three-dimensional side-chain patterns: new examples of convergent evolution J Mol Biol 1998 13 5 1211 27 10.1006/jmbi.1998.1844 9642096

[B10] Chen BY Fofanov VY Bryant DH Dodson BD Kristensen DM Lisewski AM Kimmel M Lichtarge O Kavraki LE The MASH pipeline for protein function prediction and an algorithm for the geometric refinement of 3D motifs Journal of Computational Biology 2007 13 6 791 816 10.1089/cmb.2007.R017 17691895

[B11] Bryant DH Moll M Chen BY Fofanov VY Kavraki LE Analysis of substructural variation in families of enzymatic proteins with applications to protein function prediction BMC Bioinformatics 2010 13 242 10.1186/1471-2105-11-242 20459833 PMC2885373

[B12] Dundas J Adamian L Liang J Structural signatures of enzyme binding pockets from order-independent surface alignment: a study of metalloendopeptidase and NAD binding proteins Journal of Molecular Biology 2011 13 5 713 729 http://www.ncbi.nlm.nih.gov/pubmed/21145898 10.1016/j.jmb.2010.12.005 21145898 PMC3061237

[B13] Chen BY Fofanov VY Bryant DH Dodson BD Kristensen DM Lisewski AM Kimmel M Lichtarge O Kavraki LE Geometric Sieving: Automated Distributed Optimization of 3D Motifs for Protein Function Prediction Proceedings of The Tenth Annual International Conference on Computational Molecular Biology (RECOMB 2006) 2006 500 515

[B14] Stark A Sunyaev S Russell RB A Model for Statistical Significance of Local Similarities in Structure J Mol Biol 2003 13 1307 1316 10.1016/S0022-2836(03)00045-7 12595245

[B15] Fofanov V Chen B Bryant D Moll M Lichtarge O Kavraki L Kimmel M A statistical model to correct systematic bias introduced by algorithmic thresholds in protein structural comparison algorithms 2008 IEEE International Conference on Bioinformatics and Biomeidcine Workshops 2008 1 8

[B16] Chen BY Honig B VASP: a volumetric analysis of surface properties yields insights into protein-ligand binding specificity PLoS Comput Biol 2010 13 8 pii: e1000881 20814581 10.1371/journal.pcbi.1000881 PMC2930297

[B17] Chen B Bandyopadhyay S A Statistical Model of Overlapping Volume in Ligand Binding Cavities Proceedings of the Computational Structural Bioinformatics Workshop (CSBW 2011) 2011 424 31

[B18] Chen B Bandyopadhyay S VASP-S: A Volumetric Analysis and Statistical Model for Predicting Steric Influences on Protein-Ligand Binding Specificity Proceedings of 2011 IEEE International Conference on Bioinformatics and Biomedicine (BIBM) 2011 22 9

[B19] Pasternak O Biesiadka J Dolot R Handschuh L Bujacz G Sikorski MM Jaskolski M Structure of a yellow lupin pathogenesis-related PR-10 protein belonging to a novel subclass Acta Crystallogr D Biol Crystallogr 2005 13 99 107 10.1107/S0907444904028173 15608381

[B20] Godshall B Chen B Improving accuracy in binding site comparison with homology modeling Bioinformatics and Biomedicine Workshops (BIBMW), 2012 IEEE International Conference on: 4-7 October 2012 2012 662 669 10.1109/BIBMW.2012.6470291

[B21] Koehl P Levitt M A brighter future for protein structure prediction Nature Structural Biology 1999 13 108 111 10.1038/5794 10048917

[B22] Baker D Sali A Protein structure prediction and structural genomics Science's STKE 2001 13 5540 93 11588250 10.1126/science.1065659

[B23] Yang AS Honig B An integrated approach to the analysis and modeling of protein sequences and structures. I. Protein structural alignment and a quantitative measure for protein structural distance J Mol Biol 2000 13 3 665 78 10.1006/jmbi.2000.3973 10966776

[B24] Moll M Bryant DH Kavraki LE The LabelHash algorithm for substructure matching BMC Bioinformatics 2010 13 555 10.1186/1471-2105-11-555 21070651 PMC2996407

[B25] Shatsky M Shulman-peleg A Nussinov R Wolfson HJ Recognition of Binding Patterns Common to a Set of Protein Structures Lect Notes Comput Sc 2005 13 440 455 10.1007/11415770_33

[B26] Schmitt S Kuhn D Klebe G A NewMethod to Detect Related Function Among Proteins Independent of Sequence and Fold Homology J Mol Biol 2002 13 2 387 406 10.1016/S0022-2836(02)00811-2 12381328

[B27] Berman HM Westbrook J Feng Z Gilliland G Bhat TN Weissig H Shindyalov IN Bourne PE The Protein Data Bank Nucleic Acids Res 2000 13 235 42 10.1093/nar/28.1.235 10592235 PMC102472

[B28] Shatsky M Nussinov R Wolfson HJ FlexProt: alignment of flexible protein structures without a predefinition of hinge regions J Comput Biol 2004 13 83 106 10.1089/106652704773416902 15072690

[B29] Salem S Zaki M Bystroff C FlexSnap: Flexible Non-sequential Protein Structure Alignment Algorithms for Molecular Biology 2010 13 12 10.1186/1748-7188-5-12 20047669 PMC2846951

[B30] Ye Y Godzik A Multiple flexible structure alignment using partial order graphs Bioinformatics 2005 13 10 2362 9 10.1093/bioinformatics/bti353 15746292

[B31] Xiang Z Honig B Extending the accuracy limits of prediction for side-chain conformations1 Journal of molecular biology 2001 13 2 421 430 10.1006/jmbi.2001.4865 11478870

[B32] Goujon M McWilliam H Li W Valentin F Squizzato S Paern J Lopez R A new bioinformatics analysis tools framework at EMBL-EBI Nucleic acids research 2010 13 suppl 2 W695 W699 20439314 10.1093/nar/gkq313 PMC2896090

[B33] Lee B Richards FM The interpretation of protein structures: estimation of static accessibility J Mol Biol 1971 13 3 379 400 10.1016/0022-2836(71)90324-X 5551392

[B34] Connolly M Solvent-accessible surfaces of proteins and nucleic acids Science 1983 13 4612 709 713 10.1126/science.6879170 6879170

[B35] Nayal M Honig B On the Nature of Cavities on Protein Surfaces: Application to the Identification of Drug-Binding Sites Proteins Struct Funct Genet 2006 13 892 906 10.1002/prot.20897 16477622

[B36] Chen BY Bandyopadhyay S Modeling regionalized volumetric differences in protein-ligand binding cavities Proteome Science 2012 13 Suppl 1 S6 10.1186/1477-5956-10-S1-S6 22759583 PMC3390949

[B37] Schaer J Stone M Face traverses and a volume algorithm for polyhedra Lect Notes Comput Sc 1991 13 290 297

[B38] Chen BY Bandyopadhyay S A regionalizable statistical model of intersecting regions in protein ligand binding cavities Journal of Bioinformatics and Computational Biology 2012 13 3 1242004 10.1142/S0219720012420048 22809380

[B39] Rakus JF Fedorov AA Fedorov EV Glasner ME Hubbard BK Delli JD Babbitt PC Almo SC Gerlt JA Evolution of enzymatic activities in the enolase superfamily: L-rhamnonate dehydratase Biochemistry 2008 13 38 9944 54 10.1021/bi800914r 18754693 PMC2562705

[B40] Saraste M Sibbald P Wittinghofer A The P-loop-a common motif in ATP-and GTP-binding proteins Trends in biochemical sciences 1990 13 11 430 10.1016/0968-0004(90)90281-F 2126155

[B41] Shan Y Seeliger M Eastwood M Frank F Xu H Jensen M Dror R Kuriyan J Shaw D A conserved protonation-dependent switch controls drug binding in the Abl kinase Proc Natl Acad Sci USA 2009 13 139 144 10.1073/pnas.0811223106 19109437 PMC2610013

[B42] Babbitt PC Hasson MS Wedekind JE Palmer DR Barrett WC Reed GH Rayment I Ringe D Kenyon GL Gerlt JA The enolase superfamily: a general strategy for enzyme-catalyzed abstraction of the alpha-protons of carboxylic acids Biochemistry 1996 13 51 16489 501 10.1021/bi9616413 8987982

[B43] Kühnel K Luisi BF Crystal structure of the Escherichia coli RNA degradosome component enolase J Mol Biol 2001 13 3 583 92 10.1006/jmbi.2001.5065 11676541

[B44] Schafer SL Barrett WC Kallarakal AT Mitra B Kozarich JW Gerlt JA Clifton JG Petsko GA Kenyon GL Mechanism of the reaction catalyzed by mandelate racemase: structure and mechanistic properties of the D270N mutant Biochemistry 1996 13 18 5662 9 10.1021/bi960174m 8639525

[B45] Songyang Z Carraway K Eck M Harrison S Feldman R Mohammadi M Schlessinger J Hubbard S Smith D Eng C Catalytic specificity of protein-tyrosine kinases is critical for selective signalling Nature 1995 13 6514 536 539 10.1038/373536a0 7845468

[B46] Krause D Van Etten R Tyrosine kinases as targets for cancer therapy New England Journal of Medicine 2005 13 2 172 187 10.1056/NEJMra044389 16014887

[B47] Deininger M Buchdunger E Druker B The development of imatinib as a therapeutic agent for chronic myeloid leukemia Blood 2005 13 7 2640 2653 10.1182/blood-2004-08-3097 15618470

[B48] Liu Y Shah K Yang F Witucki L Shokat KM A molecular gate which controls unnatural ATP analogue recognition by the tyrosine kinase v-Src Bioorganic & medicinal chemistry 1998 13 8 1219 26 10.1016/S0968-0896(98)00099-6 9784863

[B49] Alaimo PJ Knight Za Shokat KM Targeting the gatekeeper residue in phosphoinositide 3-kinases Bioorganic & medicinal chemistry 2005 13 8 2825 36 10.1016/j.bmc.2005.02.021 15781393

